# A hybrid chimeric system for versatile and ultra-sensitive RNase detection

**DOI:** 10.1038/srep09558

**Published:** 2015-04-01

**Authors:** Stefano Persano, Giuseppe Vecchio, Pier Paolo Pompa

**Affiliations:** 1 Istituto Italiano di Tecnologia, Center for Bio-Molecular Nanotechnologies@UniLe, Via Barsanti, 73010 Arnesano (Lecce), Italy; 2 Università del Salento, Via Provinciale Monteroni, 73100 Lecce, Italy

## Abstract

We developed a new versatile strategy that allows the detection of several classes of RNases (i.e., targeting ss- or ds-RNA, DNA/RNA hetero-hybrid or junctions) with higher sensitivity than existing assays. Our two-step approach consists of a DNA-RNA-DNA chimeric Hairpin Probe (cHP) conjugated to magnetic microparticles and containing a DNAzyme sequence in its terminal region, and molecular beacons for fluorescence signal generation. In the first step, the digestion of the RNA portion of the cHP sequences in presence of RNases leads to the release of multiple copies of the DNAzyme in solution. Then, after magnetic washing, each DNAzyme molecule elicits the catalytic cleavage of numerous molecular beacons, providing a strong amplification of the overall sensitivity of the assay. We successfully applied our approach to detect very low concentrations of RNase A, *E. coli* RNase I, and RNase H. Furthermore, we analyzed the effect of two antibiotics (penicillin and streptomycin) on RNase H activity, demonstrating the applicability of our strategy for the screening of inhibitors. Finally, we exploited our system to detect RNase activity directly in crude biological samples (i.e., blood and saliva) and in cell culture medium, highlighting its suitability as cheap and sensitive tool for the detection of RNase levels.

Ribonucleases (RNases) are a class of ubiquitous enzymes that play an important role in several biological processes such as gene expression and regulation, genome replication and maintenance, host defense, stress response, and viral strategies of infection^1^. RNases also show intrinsic cytotoxic activity that includes RNA cleavage, leading to inhibition of protein synthesis and apoptosis^2^,^3^, such as in response to accumulation of misfolded proteins within the endoplasmic reticulum^4^,^5^,^6^. The intrinsic cytotoxicity of RNases is evoking medical interest, since such enzymes could be used, alone or conjugated to ligands or antibodies, as non-mutagenic therapeutic agents for cancer treatment^7^,^8^. On the other hand, RNase contaminations represent a major problem in any experiments involving RNA, since they may degrade precious samples and invalidate the experimental outcomes. Hence, the development of rapid, robust and low-cost methods for RNases detection is highly desired, in order to improve existing methods that are typically laborious, time consuming, expensive, and low-sensitive (i.e., gel electrophoresis, radioisotope-labeled substrates based system, acid soluble release of RNA fragment, etc.)^9^,^10^. For these reasons, several innovative approaches were recently proposed in the literature to facilitate RNase detection. These are based on the detection of fluorescent^11^,^12^,^13^,^14^,^15^,^16^ or electrochemical^17^,^18^ signals, colorimetric changes^19^,^20^,^21^, or surface plasmon resonance^22^. In this context, we developed a new versatile RNase detection assay based on a hybrid chimeric fluorescent system that allows detecting a wide range of RNases with unprecedented sensitivity.

As schematized in Figure 1, our RNase assay is based on a two-step detection strategy, which exploits a chimeric Hairpin Probe (cHP) conjugated to magnetic microparticles (MMP). The cHP sequence was designed in order to contain a 3′-biotinylated DNA portion for the immobilization onto streptavidin-coated magnetic microparticles (MPP), a RNA sequence that represents the substrate both for generic RNases and for the junction ribonuclease activity of RNase H, and a DNAzyme sequence (the complete cHP sequence is reported in Table S1). In the first step, RNase recognizes and degrades the RNA strands of cHPs, inducing the release of the DNAzyme in solution. The unreacted MMP-cHPs complex is then removed by magnetic washing; this also allows an increase of the sensitivity of the assay, thanks to the reduction of the background signal caused by the spontaneous degradation of the undigested RNA portion of the cHPs^23^,^24^. In the second step, the released DNAzyme can exploit its full catalytic activity, digesting the molecular beacons that, in turn, produce a strong fluorescent signal. Hence, the overall strategy benefits from a cascading amplification, in which each RNase/DNAzyme processes a large number of substrate molecules (a single RNAse thus produces n^2^ fluorescence events, Fig. 1b). Remarkably, our system requires very low volumes of testing solution (1–4 μL) and of total solutions for biochemical reactions (ca. 40 μL). The fluorescence signals may be monitored by a simple fluorescence plate reader. All these characteristics make our approach very sensitive, fast, and cost-effective.

We first used our assay strategy to demonstrate its applicability in the detection of different RNase contaminations. RNases are widely produced in living organisms, (e.g., they are present in flaked skin and hair, in fluids such as tears, saliva, mucus, and perspiration)^25^, so they can represent a serious problem for the contamination and degradation of RNA samples in molecular biology (i.e., microarray studies, real-time PCR, Northern blots, or cDNA cloning). We used our assay to detect different classes of RNases, namely RNase A, RNase I, and RNase H. We started our studies with RNase A, a well-established model to evaluate the sensitivity of RNase assays^26^, and largely used as positive control in commercial RNase contamination detection kits. As reported in Figure 2, the experimental results show that our strategy allows sensitive detection of RNase A down to 0.85 pg after just 30 min of DNAzyme activity. However, using a longer incubation time (2 h), the sensitivity increases to 0.017 pg (corresponding to ~3 × 10^−7^ U/mL) of RNase A (Figure 2, inset).

We then tested *E. coli* RNase I. This ribonuclease degrades single-stranded RNA by cleaving between all dinucleotide pairs^27^. As shown in Figure S1, the versatility of our assay strategy enables also the detection of this class of RNases, with a detection limit as low as 0.01 U/mL in 20–30 min.

Finally, we tested RNase H. RNase H is a particular member of RNases family, which specifically hydrolyzes the RNA strand of RNA:DNA hybrid, playing a critical role in several cellular processes including DNA replication, DNA repair and transcription^1^,^28^,^29^. Moreover, as a highly conserved damage repair protein, RNase H can cleave RNA-DNA junctions in Okazaki fragment, processing through its junction ribonuclease (JRNase) activity^30^. As reported in Figure S2, the particular design of the cHP sequence allows us to evaluate the JRNase activity of RNase H with a limit of detection of 0.1 U/mL. Overall, the sensitivity of our assay is typically higher than previously reported methods^15^,^16^. The limits of detection for RNase A, RNase I, and RNase H were confirmed by calculation following the IUPAC definition (doi:10.1351/goldbook).

Our assay can be also employed for quantitative measurement of RNases activity. As a proof-of-concept, in Figure S3 and S4, we reported the quantification curve for RNase H and RNase A, respectively. The very good fitting of experimental points (R^2^ is close to 1.0) denotes the reliability of our assay to calculate RNases concentrations.

Since RNase H has been found to be associated with retroviral reverse transcriptase (e.g., in Human Immunodeficiency Virus, Hepadnaviruses, murine leukemia virus, etc.), it is becoming a new potential target for viral inhibitors, representing an exciting possibility for developing new anti-viral therapeutics^28^,^31^,^32^,^33^,^34^. We tested the potential of our approach for the screening and testing of new anti-viral drugs. We analyzed the effect of two antibiotics that were found to have inhibitory effects on RNase H, namely streptomycin and penicillin (Figure 3). In particular, we observed a strong inhibition of RNase H enzymatic activity in presence of streptomycin, in line with previous reports in which the effects of drugs on RNase H activity were evaluated from the initial velocity variation^13^. On the other hand, penicillin induced only a very small effect on RNase H even at the highest concentration (8 mM). This is in disagreement with published data^13^, although it may be possible that such discrepancy arises from the different source of RNase H used in our work, provided that slight modifications in the enzyme molecular structure can strongly impact enzyme activity and sensitivity to inhibitors^35^,^36^. Overall, experimental data indicate that our hybrid assay allows to evaluate the effects of inhibitors with high sensitivity, representing an interesting tool for testing and screening of anti-viral drugs.

RNases are also arousing medical interests, potentially representing novel biomarkers for some human diseases^2^,^4^,^7^,^8^,^16^,^37^,^38^,^39^,^40^,^41^,^42^,^43^,^44^. In fact, variations of RNase levels were found to be related to several neoplastic diseases (e.g., pancreatic, ovarian, uterus, gastric, and colon cancer, myelogenous Leukemia, etc.)^16^,^37^,^38^,^39^,^40^,^41^,^42^,^44^. Moreover, the saliva RNase level has been proposed as an important biomarker for several diseases, such as cystic fibrosis^43^. Here, we evaluated the applicability of our strategy to detect RNase levels directly in biological fluids and cell culture medium. Notably, we were able to detect RNases activity in freshly collected human blood and saliva (Figure 4). In these experiments, our assay required very small quantity of biological samples (1 μL drops of saliva and blood were further diluted 1:25). As shown in Figure 4A, the RNase activity detected in both biological fluids is very high with respect to the control. In particular, the RNase activity detected in the blood was twice than in saliva. This result is in agreement previously published reports^45^ and has also been validated using commercially available kits.

RNase activity was also detected in serum-free cell medium (Figure 4B). The fluorescence signal detected in untreated MCF7 cell was about 50% higher than the control (i.e. in absence of cells), while the fluorescence measured in cells treated with neomycin was significantly lower and comparable to the control. The RNase activity reduction observed upon treatment with neomycin is due to the inhibition of protein synthesis.

In conclusion, we have shown an innovative and versatile strategy for the detection of RNases activity, based on a hybrid chimeric probe, allowing the highly sensitive analysis of different classes of RNases, such as RNase A, RNase I and the junction activity of RNase H. These characteristics, coupled with the small volume of samples needed by our approach, make our assay suitable for future implementations, such as for low-cost Lab-On-a-Chip and high-throughput screening applications.

## Methods

The oligonucleotides used in this work (Table S1) were obtained by Integrated DNA Technologies (IDT, Coralville, Iowa, USA). Streptavidin-coated magnetic microparticles (MMPs) with a diameter of 1 μm were purchased from Invitrogen™ (Thermo Fisher Scientific Inc., Waltham, MA, USA). *Escherichia coli* RNase I (10 U/μL), and 10× TNE buffer (100 mM Tris-HCl, 1 M NaCl, and 10 mM EDTA, pH 7.5) were obtained from Epicentre (Madison, Wisconsin, USA). Recombinant RNase A (1 mg/mL) was received from Ambion® (Thermo Fisher Scientific Inc., Waltham, MA, USA). Recombinant *Escherichia coli* RNase H (1.5 U/μL) was ordered from Promega (Madison, Wisconsin, USA). Proteinase K and all DNase/RNase-free reagents used to prepare the RNase H buffer (50 mM Tris-HCl, 100 mM KCl, 9 mM MgCl_2_, 1 mM DTT, 20 μg/mL BSA, pH 7.5) were purchased from Sigma-Aldrich. In order to minimize environmental RNase contaminations, all the reagents used in this work were guaranteed RNase-free, the working area was cleaned with RNase ZAP Decontamination Solution (Sigma Aldrich). As MMPs were not supplied in RNase-free solutions, we performed washing steps with DEPC-treated solutions, according to the manufacturer's instructions.

### Preparation of the MMP-cHP complex

The chimeric Hairpin Probe (cHP) stock (10 μM) was heated at 95°C for 5 minutes and then slowly cooled (0.1°C sec^−1^) at room temperature to allow the correct folding of the oligonucleotide sequence. The biotinylated cHP sequences were then conjugated to streptavidin-coated MMPs following manufacturer's instructions. Briefly, MMPs (10 mg/mL) were washed three times in RNase-free buffer to remove the preservative storage buffer. Then, MMPs were suspended in RNase-free buffer and incubated at final concentration of 5 mg/mL, with a 2 μM solution of cHP for 10 minutes at 4°C, and 10 minutes at 25°C to allow biotin-streptavidin conjugation. Finally, the obtained MMP-cHP complex was washed three times with RNase-free buffer to remove the unbound cHPs. In order to confirm the successful conjugation of the cHPs on the MMP surface, the MMP-cHPs complex was further characterized by Zeta-potential and UV/vis analyses. The Z-pot measurements were performed with a Zetasizer Nano ZS90 (Malvern, USA) (Fig. S5). The binding capacity of MMPs was estimated by DNA/RNA quantification using NanoDrop 2000 spectrophotometer. Briefly, after the MMP-cHPs conjugation and washing steps as described above, the immobilized cHPs molecules were released from the MMPs, following the manufacturer's instructions (2 min at 90°C in 10 mM EDTA, pH 8.2 with 95% formamide), and quantified through UV-vis measurements after MMPs removal. We obtained a value of 2.8 × 10^4^ chimeric probes per particle.

### Generic RNase assay protocol

The MMP-cHP complex was used at a final concentration of 1.25 mg/mL, in RNase buffer at final volume of 40 μL. The first step of the assay was carried out at different time, buffer and temperature conditions, depending on the tested RNase (see below). After enzymatic RNA degradation, the MMPs were removed by applying a magnetic field, and the supernatant, containing the released DNAzyme, was collected and used in the second step. The fluorescence signal was obtained by digestion of the molecular beacons (MB) by the DNAzyme. The MB digestion was carried out using 10 μL of supernatant containing the released DNAzyme mixed to 10 μL of a solution containing 1 μM of MB and 10 mM of MgCl_2_. This reaction was carried out at 37°C using a TECAN Infinite® M200 Pro plate reader; fluorescence (Ex/Em = 485/522) was monitored every 10 min for 2 hrs.

### RNase A and *E. coli* RNase I activity assay

Different amounts of RNase A (0.0085 to 170 pg) and *E. coli* RNase I (0.001 to 100 U/mL) were added in 1× TNE buffer (10 mM Tris-HCl, 100 mM NaCl, and 1 mM EDTA, pH 7.5) containing 1.25 mg/mL of MMP-cHPs conjugate (40 μL total solution). After incubation for 30 minutes at 37°C, the excess of MMP-cHP complex was removed by magnetic washing.

### RNase H activity assay

The RNase H activity was tested using different concentration of RNase H (0.05–25 U/mL) in 40 μL of RNase H buffer (50 mM Tris-HCl, 100 mM KCl, 9 mM MgCl_2_, 1 mM DTT, 20 μg/mL BSA, pH 7.5) containing 1.25 mg/mL of MMP-cHPs conjugates. The enzymatic reaction was carried at 25°C for 20 minutes, then the undigested MMP-cHP complex was removed by magnetic washing and 10 μL of supernatant were collected from each sample.

### RNase H Inhibition Assay

The RNase H enzyme was pre-incubated for 10 minutes at 25°C in the absence (negative control) or presence of various concentrations (0.5–8 mM) of penicillin and streptomycin sulphate. Then, MMP-cHP complex was added to pre-incubated RNase H (final volume 40 μL, final concentration of MMP-cHP complex 1.25 mg/mL), and incubated for 10 minutes at 25°C. Fluorescence signals were collected after 40 minutes.

### Cell culture and RNase activity in cell medium

MCF7 cells (derived from primary tumor, human invasive breast ductal carcinoma ATCC® HTB-22™) were grown in DMEM medium (Gibco) supplemented 10% FBS (Gibco) + 2 mM L-Glutamine (Sigma Aldrich), 1% PenStrep. Cells were seeded at density of 10^5^ cells/ml in 96 well plate, grown for 24 h, and then starved for 12 h in serum free. For neomycin treatment, cells grown for 18 h in complete medium were incubated with 1 mg/mL of neomycin for 6 h, then transferred in DMEM serum free medium for other 12 h. Finally, cell cultures were centrifuged 10 min at 500 g, and 10 μL of each supernatant was used for RNase assay. As control, we tested the RNase activity in serum free DMEM medium.

### RNase activity in biological specimens

Few microliters of blood and saliva were freshly collected and immediately diluted 1:25 with 1× TNE buffer. For RNase activity assay, 1 μL of diluted samples was mixed to 39 μL of 1× TNE buffer containing 1.25 mg/mL of MMP-cHP complexes and incubated for 10 min at 25°C. After the magnetic washing, 10 μL of supernatant was collected and mixed with 10 μL molecular beacon solution (1 μM of MB and 10 mM of MgCl_2_). Fluorescence intensity emission was monitored every 10 minutes at Ex/Em = 485/522 λ using a TECAN Infinite® M200 Pro plate reader. In order to confirm that increased fluorescence observed in our assay is solely due to DNAzyme released by RNase activity and to exclude any interferences (for example due to the activity of proteinases in saliva samples), the MMP-cHP conjugates were incubated for 10 minutes at 30°C with Proteinase K (5 μM of Proteinase K, 50 mM Tris-HCl, and 5 mM CaCl2, pH 8.0). Furthermore, we incubated the diluted blood sample with solution of molecular beacon to exclude any interference on the fluorescence emission. In both case, results were comparable to the negative controls, confirming that biotin-streptavidin complex is resistant to proteinase digestion and blood is too diluted to interfere with fluorescence signals.

The experimental results obtained with our hybrid assay were also validated through direct comparison with a commercially available kit (RNase alert from Life Technologies).

### Limit of Detection

The fluorescence intensity corresponding to the LoD was also estimated in accordance with standard IUPAC definition (doi:10.1351/goldbook.L03540) as the 10 times the standard deviations of the blank signal (5 independent measurements).

## Author Contributions

S.P. performed the experiments. S.P., G.V. and P.P.P. designed the experiments, analyzed the data, and wrote the manuscript.

## Supplementary Material

Supplementary Information Supplementary Information

## Figures and Tables

**Figure 1:**
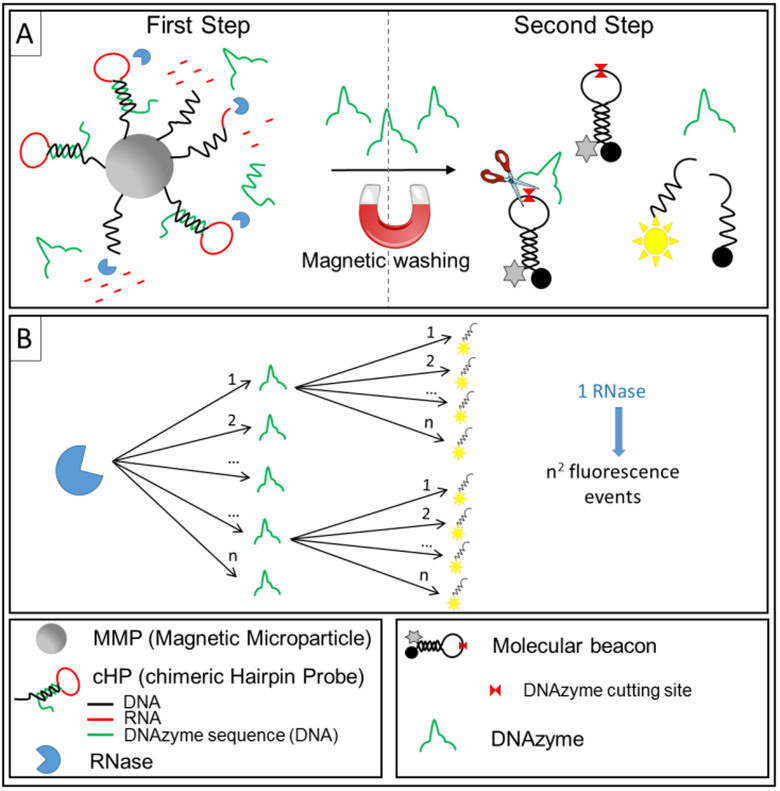
(A) Schematic illustration of the two-step RNase assay. The DNA-RNA-DNA chimeric Hairpin Probe (cHP) was immobilized by biotin-streptavidin interaction onto Magnetic Microparticles (MMP) in order to obtain the MMP-cHPs complex. In the first step, the digestion of the RNA portion of cHPs by RNase allows the release of DNAzyme. After magnetic washing, the released DNAzyme is added to FAM/Dabcyl molecular beacon. In the second step, the catalytic activity of the DNAzyme on the molecular beacons generate fluorescence signal. (B) Scheme of the amplification strategy.

**Figure 2:**
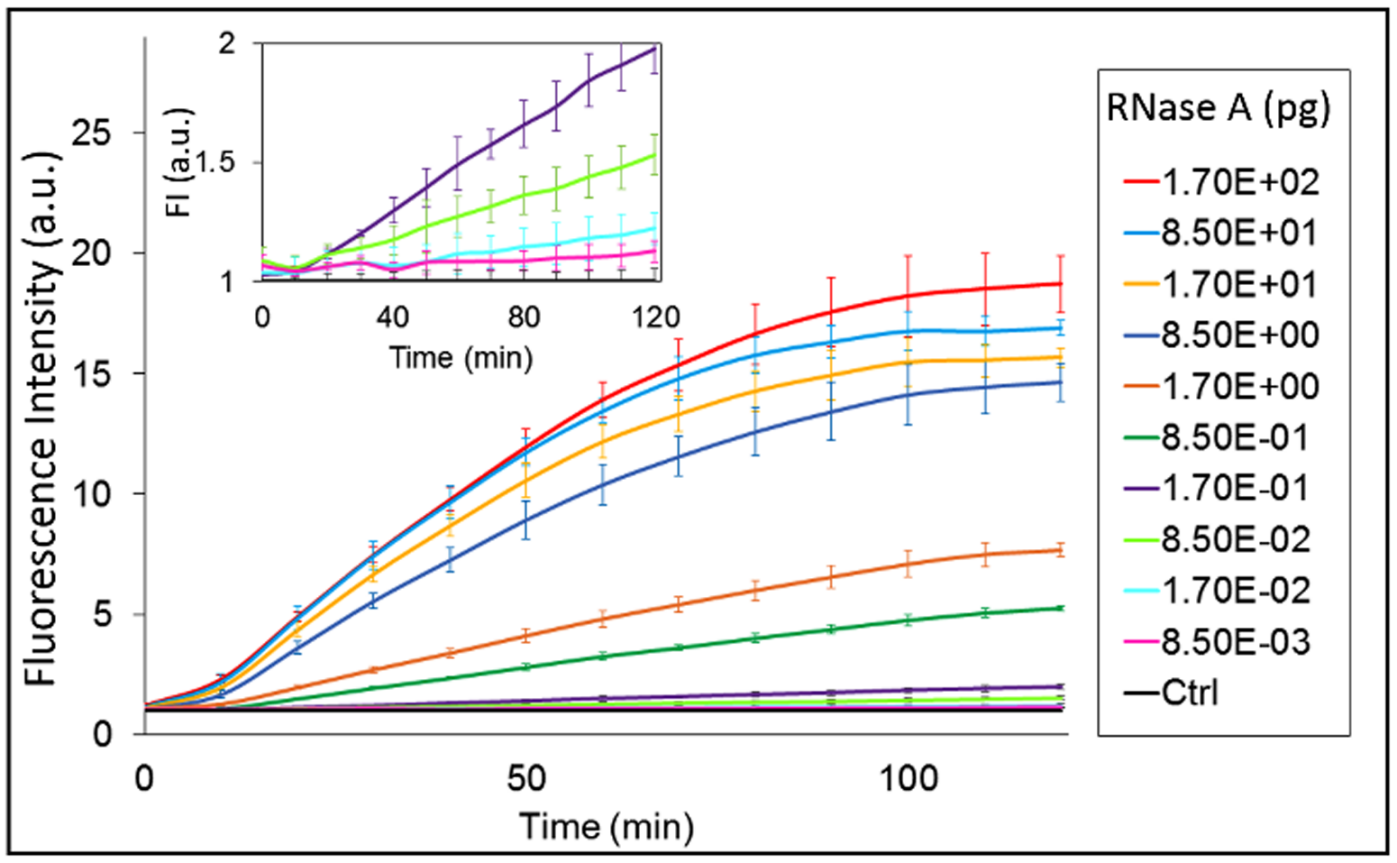
Time-dependent normalized fluorescence intensity (F/F0) at 522 nm upon incubation of our hybrid probe with different RNase A amounts (0.0085–170 pg) for 30 min at 37°C. The first step duration was fixed at 30 min. Inset shows a zoom of the kinetic curves at the lowest enzyme amounts. Error bars represent the standard deviation (SD) calculated from triplicate experiments.

**Figure 3:**
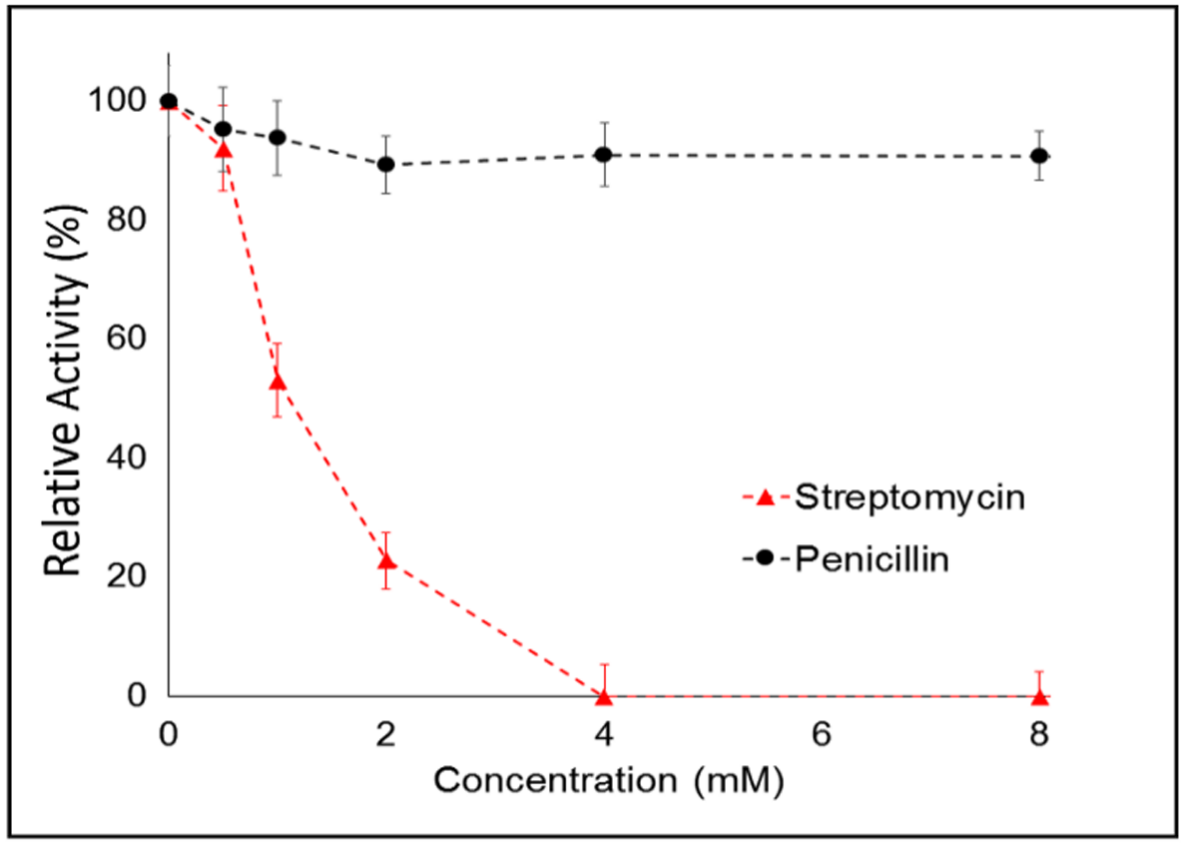
Dose-response curve showing inhibition of RNase H activity by penicillin and streptomycin. The 100% of signal corresponds to the activity of 10 U/mL of RNase H (20 min at 25°C). Fluorescence values were collected after 40 min in the second step. Error bars represent the standard deviation (SD) calculated from triplicate experiments.

**Figure 4:**
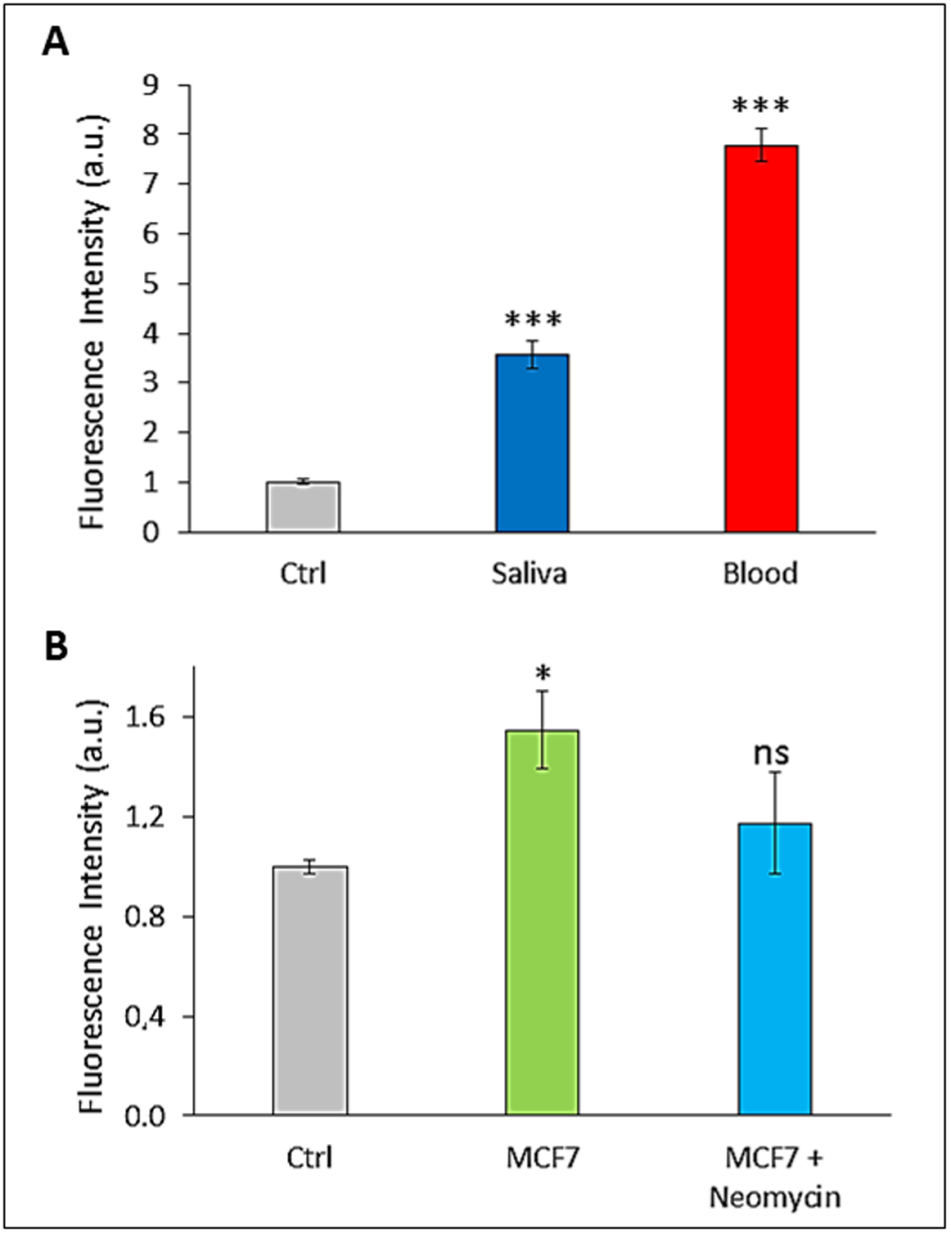
RNase activity in freshly collected biological samples and cell culture medium. Enzymatic digestion in the first step was carried out at 25°C for 10 min; fluorescence signal in the second step was collected after 60 min. (A) RNase activity in freshly collected biological samples: saliva and blood. Values were collected with a volume of 1 μl of blood and saliva diluted 1:25. (B) RNase released by MCF7 cells in serum-free medium. Control (Ctrl) measurement was obtained using the serum-free medium without cells, MCF7 refers to the medium with cells, Neomycin indicates the medium of MCF7 cells after treatment with 1 mg/mL Neomycin for 6 hours. Statistical significance was determined by t-test (* P value < 0.01; *** P value < 0.0001; ns = not significant P value > 0.05).
