# Supplementary material for: A hybrid chimeric system for versatile and ultra-sensitive RNase detection
Source: Sci Rep. 2015 Apr 1;5:9558. doi: 10.1038/srep09558 (PMC4381352; doi:10.1038/srep09558)
Supplement: Supplementary Information [file srep09558-s1.pdf]

A hybrid chimeric system for versatile and ultra-sensitive RNase detection

Stefano Persano[§‡], Giuseppe Vecchio[§], and Pier Paolo Pompa[*§]

[§] Istituto Italiano di Tecnologia, Center for Bio-Molecular Nanotechnologies@UniLe, Via Barsanti, 73010 Arnesano (Lecce), Italy.

[‡] Università del Salento, Via Provinciale Monteroni, 73100 Lecce, Italy

Supporting information

[*]corresponding author: Dr. Pier Paolo Pompa, E-mail: pierpaolo.pompa@iit.it; Fax: +39-0832-1816230; Tel: +39-0832-1816214

Name	Sequence
chimeric Hairpin Probe (cHP) (81nt)	5'- CATCTCTTCTCCGAGCCGGTCGAAATAGTGAGTrUrArArArU rUrUrGrCrArGrUrArGrArCrCrCrArGrArGrCrCTTCGACCGTTTTTT TTTTTTTTTT/Biotin/ -3'
Molecular Beacon (MB) (49 nt)	5'- /FAM/CACCACCTTTTTACTCACTATrAGGAAGAGATGTTT TTTTTTTGGTGGTG/DABCYL/ -3'

Table S1. Sequences of the oligonucleotides used in this work. In the cHP sequence, the RNA is reported in red, the DNAzyme in green, and the biotinylated DNA fragment in black. In the MB the underlined bases represent the stem sequence, while the DNAzyme substrate is reported in red. The prefix "r" denotes ribonucleosides.

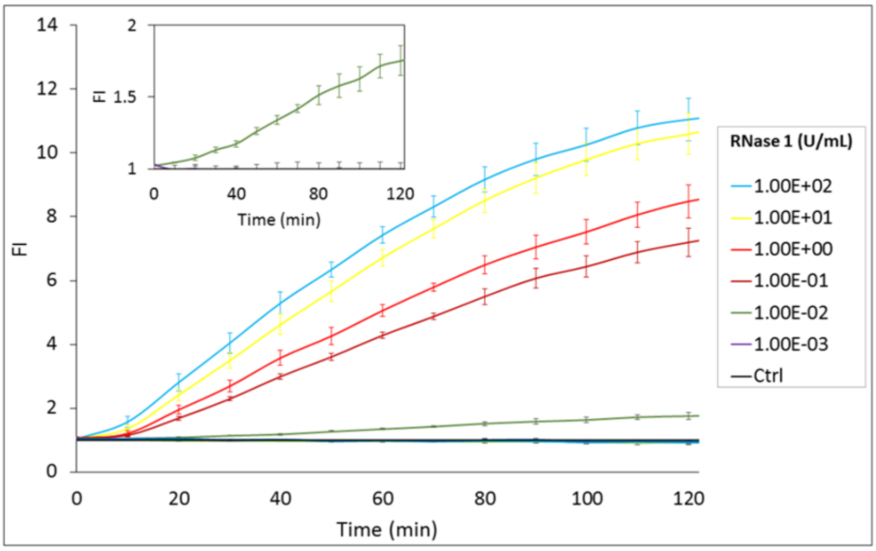

Figure S1. Time-dependent normalized fluorescence intensity (F/F0) at 522 nm upon incubation of our hybrid probe with different *E. coli* RNase I amounts (0.001-100 U/mL) for 30 min at 37 °C. Inset shows a zoom of the kinetic curves at the lowest enzyme amounts. Error bars represent the standard deviation (SD) calculated from triplicate experiments.

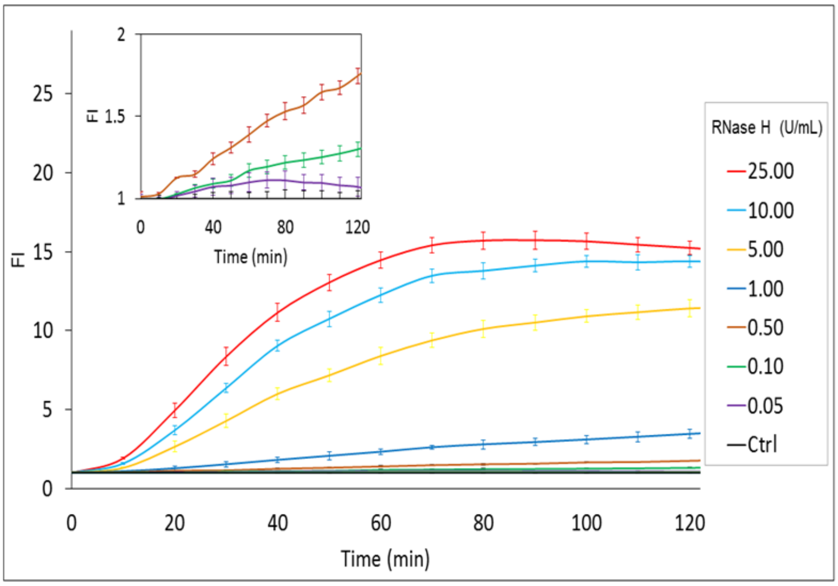

Figure S2. Time-dependent normalized fluorescence intensity (F/F0) at 522 nm upon incubation of our hybrid probe with different RNase H concentrations (0.05-25 U/mL). The fluorescence intensity emission (F/F0) was collected at 522 nm. Inset shows a zoom of the kinetic curves at the lowest enzyme amounts. Error bars represent the standard deviation (SD) calculated from triplicate experiments.

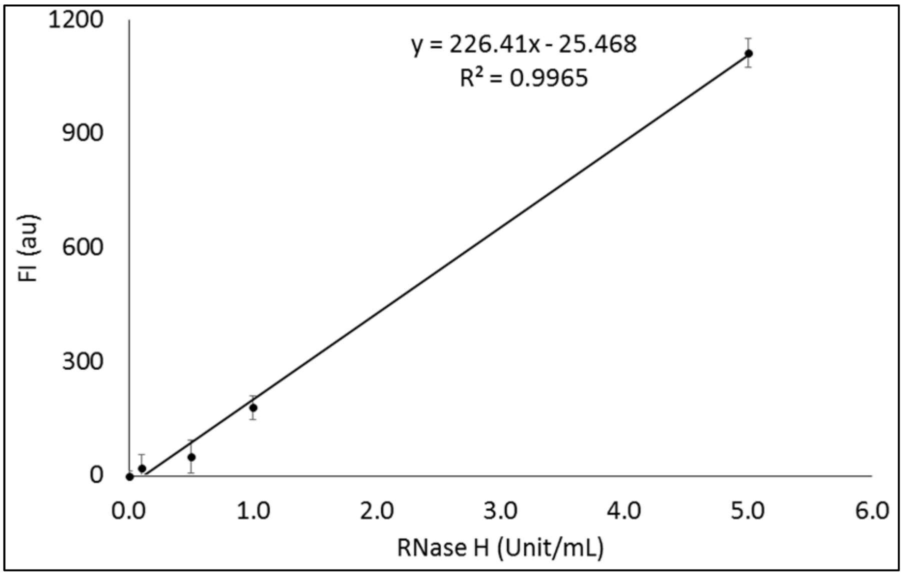

Figure S3. RNase H quantification. Data correspond to blank subtracted fluorescence signals obtained with different RNase H concentrations (0.1-5 U/mL) collected after 20 minutes of DNAzyme activity. Error bars represent the standard deviation (SD) calculated from triplicate experiments.

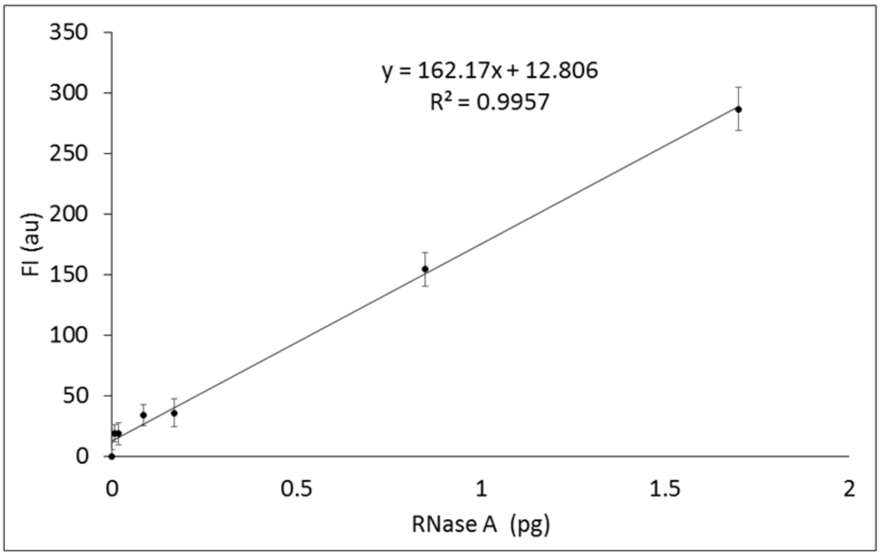

Figure S4. RNase A quantification. Data correspond to blank subtracted fluorescence signals obtained with different RNase A amounts (0.017-1.7 pg) collected after 20 minutes of DNAzyme activity. Error bars represent the standard deviation (SD) calculated from triplicate experiments.

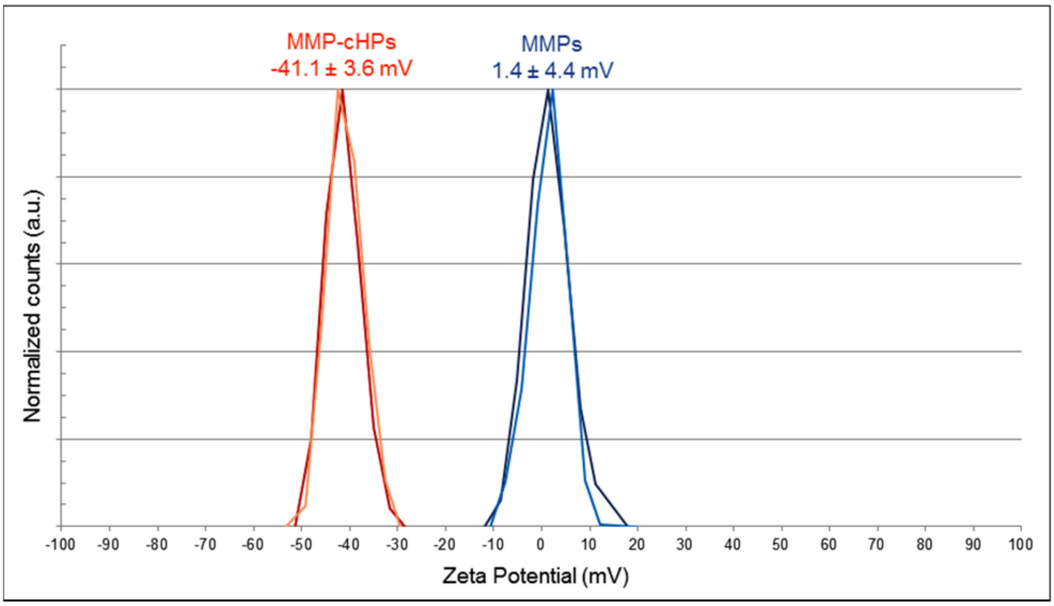

Figure S5. Zeta-potential measurements of the magnetic microparticles (MMPs) before and after their functionalization with the chimeric hairpin probes (MMP-cHPs), indicating successful bioconjugation of the beads with the probe sequences.
